# A novel method for achieving an optimal classification of the proteinogenic amino acids

**DOI:** 10.1038/s41598-020-72174-5

**Published:** 2020-09-18

**Authors:** Andre Then, Karel Mácha, Bashar Ibrahim, Stefan Schuster

**Affiliations:** 1 Chair of Bioinformatics, Matthias Schleiden Institute, University of Jena, Ernst-Abbe-Platz 2, 07743 Jena, Germany; 2 Present Address: Westernacher Solutions, Columbiadamm 37, 10965 Berlin, Germany; 3 Department of Mathematics and Natural Sciences, Centre for Applied Mathematics and Bioinformatics, Gulf University for Science and Technology, 32093 Hawally, Kuwait

**Keywords:** Chemical biology, Synthetic biology

## Abstract

The classification of proteinogenic amino acids is crucial for understanding their commonalities as well as their differences to provide a hint for why life settled on the usage of precisely those amino acids. It is also crucial for predicting electrostatic, hydrophobic, stacking and other interactions, for assessing conservation in multiple alignments and many other applications. While several methods have been proposed to find “the” optimal classification, they have several shortcomings, such as the lack of efficiency and interpretability or an unnecessarily high number of discriminating features. In this study, we propose a novel method involving a repeated binary separation via a minimum amount of five features (such as hydrophobicity or volume) expressed by numerical values for amino acid characteristics. The features are extracted from the AAindex database. By simple separation at the medians, we successfully derive the five properties volume, electron–ion-interaction potential, hydrophobicity, α-helix propensity, and π-helix propensity. We extend our analysis to separations other than by the median. We further score our combinations based on how natural the separations are.

## Introduction

The tendency to categorize and classify objects is crucial to human understanding of the surrounding world. Starting with archaic approaches such as the separation of plants into toxic and edible ones, scientific research involves classification such as in the taxonomy of organisms. We also know this from the prioritization of tasks we are confronted with in our daily work. A good classification of the fundamental elements of a system can often lead to an improved understanding of higher system behavior.

In this paper, we focus on the classification of the 20 proteinogenic amino acids, the building blocks of proteins. This helps us understand why some amino acids show a preference for occurrence in certain secondary structures of proteins^1^, develop electrostatic, hydrophobic, stacking and other interactions, or are more easily exchanged against each other in a coding sequence, which is the principle underlying substitution matrices^2,3^. Classifying amino acids is also very useful in understanding early evolution. For example, it turned out that aminoacyl tRNA synthetases can be assigned to two distinct classes. Interestingly, each class transfers exactly 10 amino acids each^4^.

**Figure 1:**
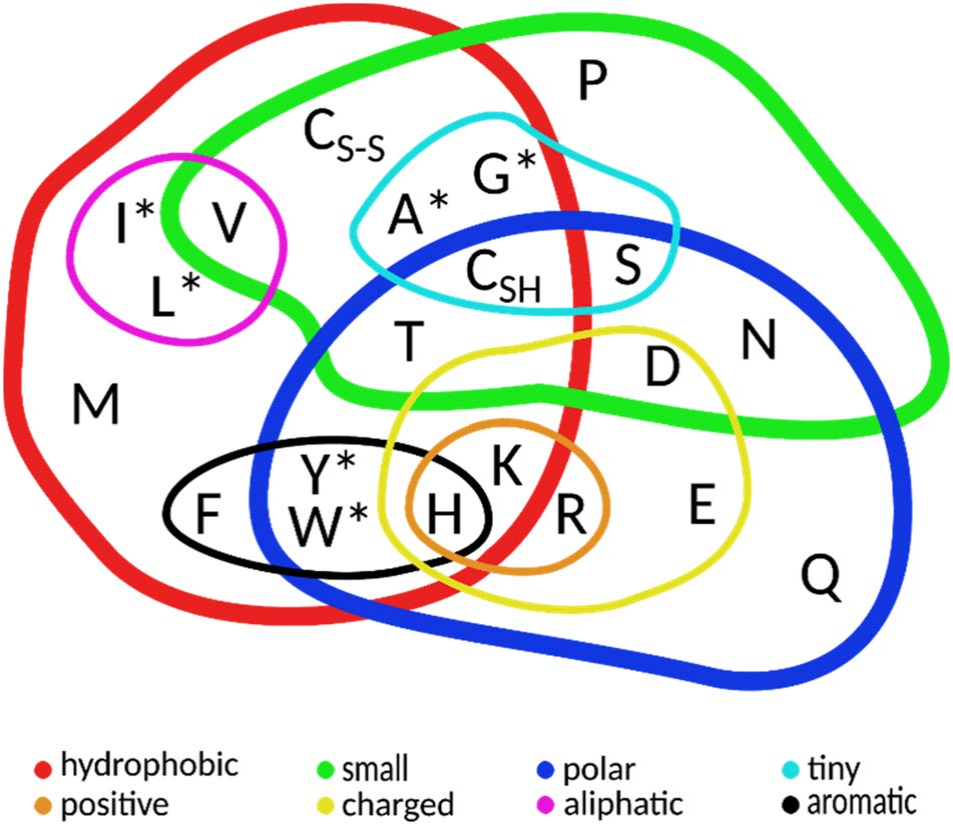
Euler diagram illustrating Taylor’s classification of the 20 amino acids (modified and extended from^5^). Amino acids are depicted by the one-letter code. Cysteine occurs twice, once in its reduced form (C_SH_) and once forming a disulfide bond (C_S-S_).

Ramsay Taylor^5^ introduced a classification scheme of eight physicochemical measures to classify the 20 amino acids and organized this scheme into an Euler diagram based on the work of Dickerson and Geis^6^ (Fig. 1). He aimed at an improved description of amino acid relatedness in protein sequence alignments. Taylor’s classification has been particularly useful in its interpretability because he only used measures easy to understand as, for example, polarity and hydrophobicity. Furthermore, the Euler diagram overall allows for a better and visually appealing understanding of the classification scheme. In later extensions of Taylor’s classification, the number of features was extended to 11, by including “proline” (a feature only shown by proline), “glycine” and “negatively charged”^7^.

Taylor’s classification, however, has two major drawbacks. First, it is non-unique in that leucine and isoleucine are in the same group, and tryptophan and tyrosine are together in one of the other groups. Moreover, it uses more than the amount of 5 features required to characterize the 20 amino acids by binary separation into unique groups, i.e. groups only containing exactly one amino acid each. Five features are sufficient because this amount can separate up to 2^5^ = 32 elements in a binary fashion, whereas four features are insufficient since 2^4^ = 16. The unnecessarily high number of features in Taylor’s classification is due to redundancy such as the features “small” and “tiny”, since every tiny amino acid is also small. To balance the mathematician’s strive for optimality and the biologist’s wish for interpretability of the features used, we aim at picking the minimum amount of five features from a list of easily interpretable features for achieving an unequivocal classification of the amino acids.

A classification fulfilling the above-mentioned criteria could also be considered as a scheme defining a unique biochemical niche for every amino acid. Thus, it serves as an approach to answering the question of whether the use of the set of proteinogenic amino acids is determined by adaptive properties or if an alternative set of amino acids might be used as well. Note that there is an enormous number of non-proteinogenic amino acids occurring in living organisms^8^, which could, alternatively, have been selected to be proteinogenic. For example, why were leucine and isoleucine selected rather than norleucine or tert-leucine, that is, the remaining two isomers of aliphatic amino acids involving four carbons in the side chain^8^? Evolution would certainly have brought about pathways for the synthesis of these alternative amino acids, since even present-day enzymes with broad substrate specificity exist in living organisms allowing for the concomitant biosynthesis of norleucine^9^ and the synthesis of tert-leucine is feasible in some bacteria living in mutualism with sponges^10^ and in engineered organisms^11^.

The question why those particular amino acids have been selected during evolution has been tackled from a multitude of different perspectives. Among them are the qualitative description of the unique functional role of every amino acid^12^ and comparing the coverage of selected physicochemical properties with a random set of amino acids^13–15^. Both of them have their limitations. The first approach can principally provide a unique role for any element as the description gets more and more detailed, while the results of the second approach depend heavily on the physicochemical properties chosen and the selection of the amino acid pool the elements of the random sets are selected from.

Importantly, on identifying a minimal set of features uniquely classifying each proteinogenic amino acid, the features can be identified in the process rather than beforehand. The result could also indicate as to which amino acids should have a high priority to artificially expand the genetic code^16,17^. It should be those which occupy some of the 12 groups (32 groups formed by five features minus 20 occupied by the proteinogenic amino acids) left unoccupied so far.

In this work, we propose a novel method based on binary separation at the median of a multitude of easily interpretable features to identify an optimal classification in the sense outlined above. We recall that the median is the value separating the higher half from the lower half of a data sample. The required features were extracted from the AAindex database^18–20^. That database consists of three lists: amino acid indices (used here), amino acid mutation matrices, and amino acid pair-wise contact patterns. As each entry of the latter two lists refers to two amino acids, these lists are less suitable for our purpose. Advantageously, the data in the AAindex is structured in a manner easily accessible through web-scraping, can be downloaded and is nearly complete in the sense that for every index there exist values for all the amino acids, though a few values are missing or not defined and were then arbitrarily set to zero in AAindex (see Table S2 for a list of the selected features containing missing values).

## Methods

### Overview of the optimal classification method

From among the properties listed in AAindex, we only consider those features that are meaningful in a biological context. In the selection of features, two different approaches are reasonable. In a first approach, we only consider those features that describe each particular amino acid per se (e.g. molecular mass or dipole moment) rather than propensities for certain secondary structures, interaction strengths or the like, because the latter features are context-dependent. This approach also has the advantage that the features can be determined for non-canonical amino acids more easily than context-dependent features because, for example, secondary structures are much less known for these.

However, by this first approach, we have not found any combination of five features classifying all 20 standard amino acids in the way described above. Only when we allow for the property of mutability, which is not purely a property of an amino acid itself, we find solutions. Four combinations of features have been determined, all of which involving mutability, bulkiness and pK-a. The latter is the experimentally determined pK value of the corresponding carboxylic acid, i. e. the amino acid without its α-amino group (FAUCHÈRE, Charton et al. 1988). The remaining two features are the pK value of the carboxy group of the entire amino acid (where one can choose between two different ways of measurement) and either the radius of gyration of the side chain or the accessible surface area.

In spite of the puristic elegance of the first approach, it has the drawback that some of the obtained features are not widely used in biochemistry and protein structure prediction. In a second approach, we allow for context-dependent features such as propensities for certain secondary structures. However, we exclude Liquid Chromatography Retention Factors as those are not of direct significance in biological systems. Although the initial classification according to Taylor^5^ does not use properties corresponding to secondary structure data, we decided to include this data as it is strongly linked to the structural features of the amino acids themselves. It is widely known for example that β-branched alkyl side chains in amino acid destabilize α-helices^21^. Moreover, to guarantee the wide applicability of our classification we only included features representing basic concepts which are also graspable for undergraduate students in the life sciences. Based on these criteria, we manually selected 83 of the 566 features (Table S1).

Mathematically, the feature patterns determining the classification can be written as binary vectors. Since we want to classify the 20 canonical amino acids and five features are sufficient for that purpose, we search five vectors of length 20, with elements 0 or 1. The features should be as “independent” as possible. Sometimes, this requirement in classification tasks is expressed in that the features should be “orthogonal” to each other. However, this requirement is too strict; a sort of linear independence is sufficient. Moreover, both orthogonality and linear independence require a Euclidean space in which the vectors are embedded. However, for binary vectors, this type of space is unnecessary and inappropriate.

The relevant criterion is the following non-redundancy criterion: *n* features are mutually non-redundant, if and only if none of the resulting partition sets involves more than 2^(5−*n*)^ elements. For example, the combination of “tiny” and “small” is inappropriate because the remaining 11 amino acids cannot be uniquely classified by three features (2^3^ = 8). Although the term non-redundancy might be questionable in the case of just one feature, the above criterion can be applied also in the case *n* = 1. For example, the feature “positively charged” in Taylor’s classification is inappropriate because only three amino acids have this feature (Fig. 1). The remaining 17 amino acids cannot be uniquely classified by four features (2^4^ = 16). Also, in Taylor’s classification, some features generate sets that are subsets of others, that is, an inclusion relation. For example, each tiny amino acid is also small. Intuitively, one might be tempted to avoid inclusion relations because they appear to imply redundancy. However, this is not necessarily the case. For example, the hypothetical feature patterns (11 11 11 00 00 00 00 00 00 00) (e.g. “small”) and (11 11 11 11 11 11 11 00 00 00) (e.g. “moderate size”) form an inclusion relation. However, they separate the 20 elements in subsets of cardinalities 6 (small and moderate size), 8 (moderate size but not small) and 6 (neither small nor moderate size), so that another three features could separate them in a way that each subset involves one element only.

The aforementioned non-redundancy criterion can best be fulfilled if each vector involves about 10 ones and 10 zeroes. Therefore, for our optimal classification, we started by dividing the amino acids into two sets based on the median of a starting feature. The first set contains all the amino acids that are smaller than or equal to the median; the second set contains all those that are greater than the median. The resulting sets were further divided by the median of the next feature, applied to all 20 amino acids. Thus, the two first steps of the algorithm generate four subsets partly overlapping each other.

**Figure 2:**
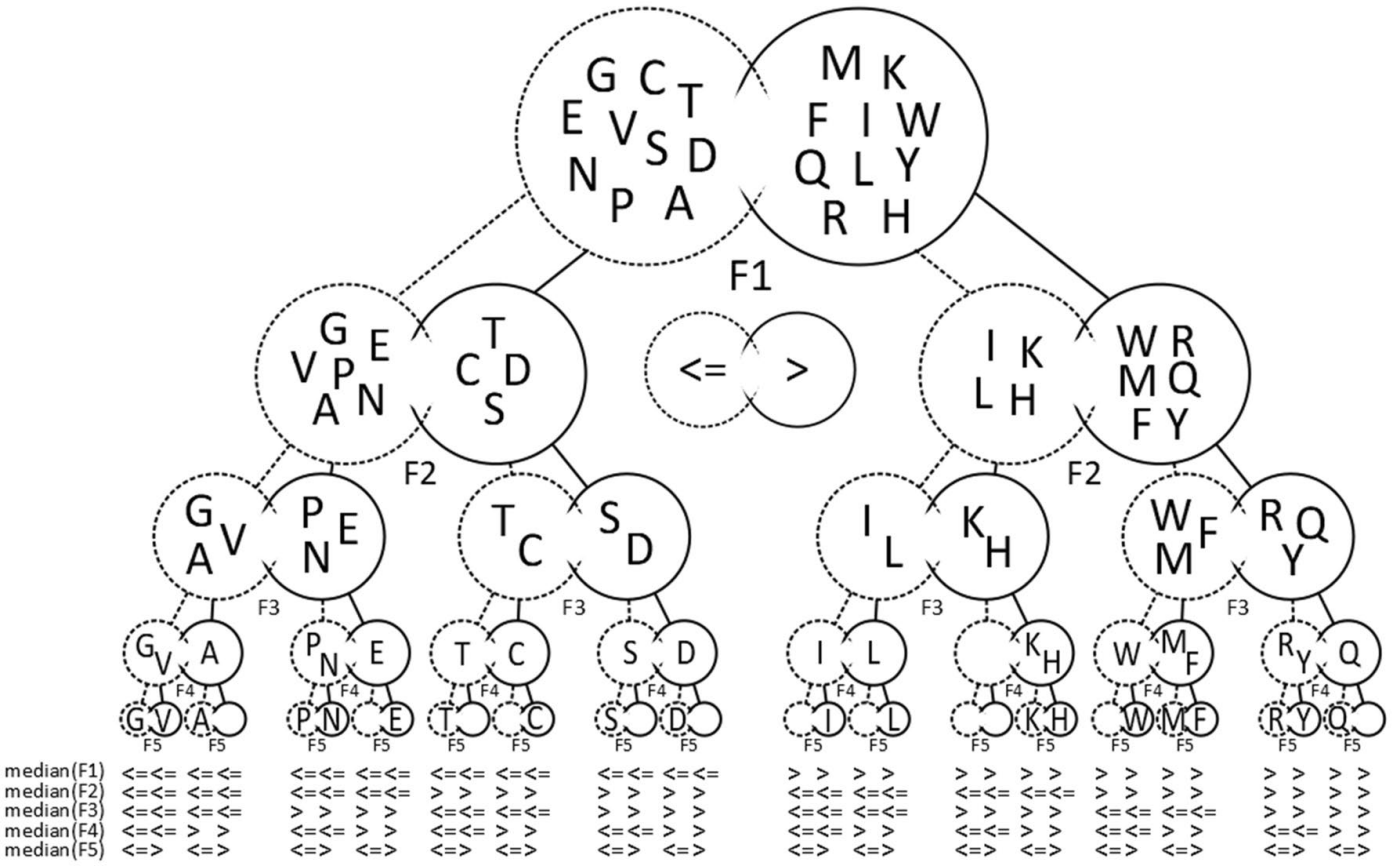
Overview of the presented classification approach. Amino acids are depicted by the one-letter code. Through repeated binary separation at the corresponding median of five features F(i) the amino acids are separated into unique groups. As five features are able to separate 32 elements, exactly 12 groups visible at the bottom of the chart remain empty. F1 = Volume, F2 = Electron–ion-interaction potential, F3 = Hydrophobicity, F4 = α-helix propensity, F5 = π-helix propensity.

To keep the runtime low, this procedure only continues until a set is formed which contains a higher number of elements than what can be separated by the remaining amount of features (e.g. a set of nine elements is formed with only three more features left). The separation by the previous feature is then revoked and the next feature on the list is chosen. Once five features have successfully been used to separate the amino acids into groups containing at most one amino acid, the corresponding list of features is retrieved (see Fig. 2 for the separation process leading to one of our median-based solutions depicted as a tree graph.).

Later we also included separations other than by the median by transferring either the amino acid(s) with the lowest value(s) above the median to the lower set or the amino acid(s) with the highest value(s) below the median to the upper set. To not let the number of possible combinations explode, we only included those separations of a particular feature which separate between adjacent amino acids which show a relatively large difference in their numerical values. For a given feature we only included the two separations with the largest difference between adjacent amino acids. Beforehand though, we excluded separations which separate into groups of < 4 and > 16, as those separations can never be part of an optimal classification (see explanation above).

### Pseudocode

The following function written in pseudocode comprises the main component of our approach towards identifying optimal classification by binary separation at the median.
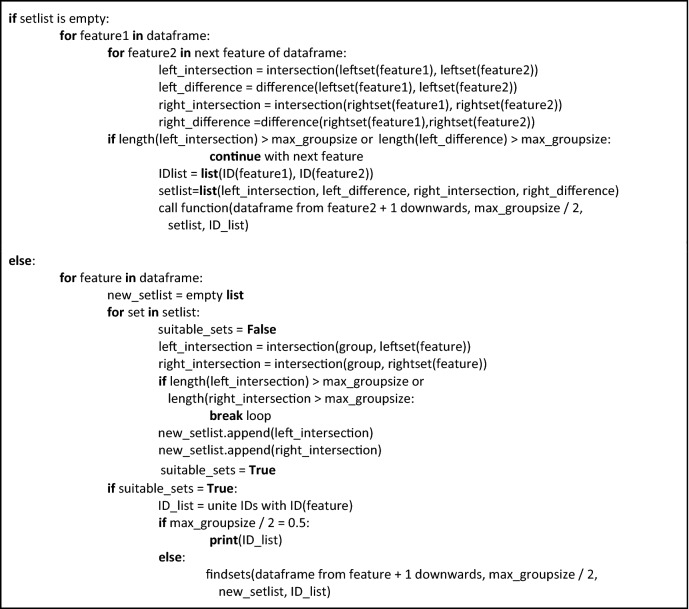


### Implementation and data analysis

The code was implemented in Python 3.6.6. Additional packages used are pandas 0.23.4 for handling and analysis of data and PyBioMed-1.0 package^22^ for accessing the AAindex data. The figures were created with Inkscape whereas the charts have been drawn with Excel 2013. The code is publicly available at https://github.com/Athen-Projects/AA_optimal_classification.

## Results and discussion

### Combinations by median based separation

**Figure 3:**
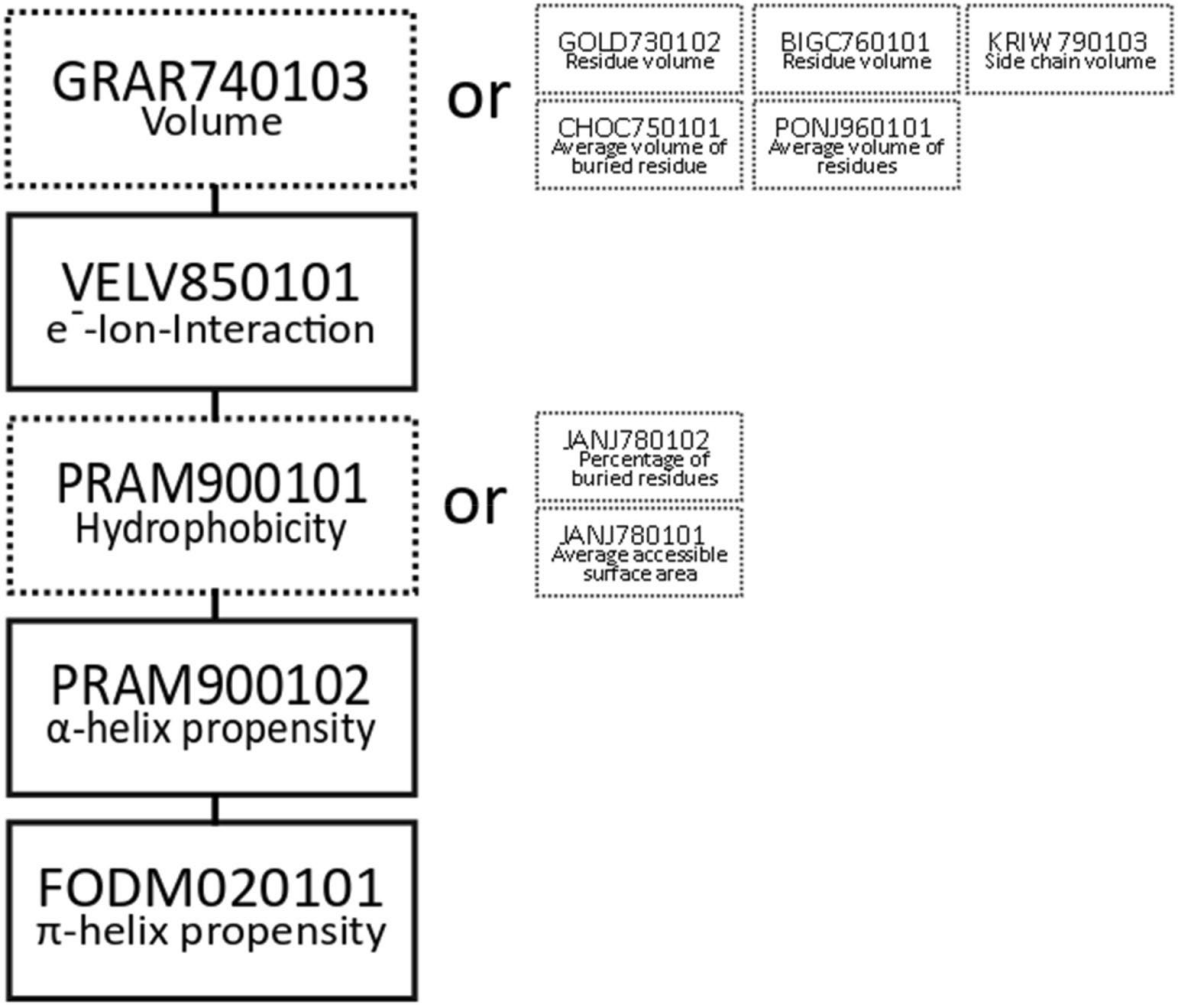
Eighteen combinations of features were found to fulfill our criteria for an optimal classification. In each box the AAindex code and a descriptive name are included. Three features occur in every of the discovered combinations (boxes with solid lines). Features in boxes with dotted lines can be substituted by one of the alternatives to their right.

**Figure 4:**
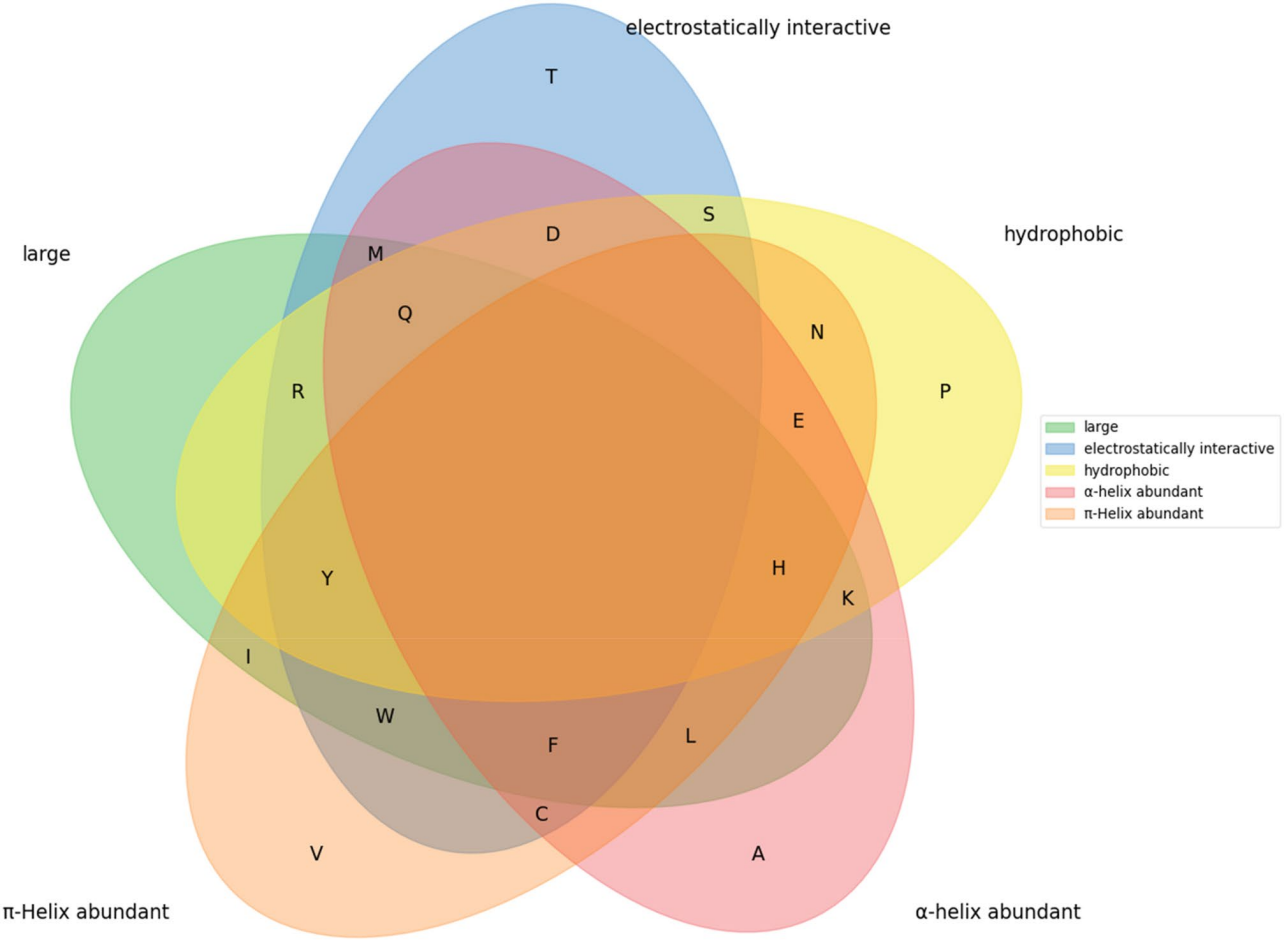
Euler diagram for the optimal classification. Glycin (G) is always separated left of the median, therefore it does not occur in the Euler diagram. The euler diagram was created with the publicly available python code from https://github.com/tctianchi/pyvenn.

By the median-based approach explained above, we were successful in finding combinations of five features each to classify the 20 standard amino acids (see Fig. 2). Each of these combinations includes the features electron–ion-interaction-potential, α-helix propensity and π-helix propensity (Fig. 3, explanation see below). Moreover, each solution required one out of six volume-related alternatives (the simplest being the amino acid volume) and one out of three hydrophobicity-related alternatives (the simplest being amino acid hydrophobicity). Thus, 18 combinations of indices through binary separation by the median were obtained. The obtained classification is also shown in an Euler diagram in Fig. 4.

Considering the solution with the simplest alternatives chosen, the first two of the indices are the volume^23^ and the hydrophobicity^24^ of the amino acids. The third is the electron–ion-interaction-potential of an amino acid^25^, which is a calculated pseudopotential based on the power of electrostatic interactions. The electron–ion-interaction-potential has been shown to be of significance for biological interactions as it correlates with carcinogenicity and similar measures^26^. The last two indices are the α-helix propensity^24^ and π-helix propensity^27^. Propensity refers to the abundance of the amino acid within the secondary structure divided by the general sequence abundance in organisms. The amino acid is therefore enriched in the corresponding secondary structure if the propensity is higher than 1.

Hydrophobicity is a very important feature in protein biochemistry. It is essential when amino acids serve to bind hydrophobic ligands or protein domains are embedded in membranes. Moreover, determining the sequence period of hydrophobic amino acids is instrumental in predicting coiled-coil structures, due to the hydrophobic core formed by these amino acids^28^. The most wide-spread period is 7 (heptad motif), like in α-keratin. Other periods can also be observed, such as 10 (dekad) or 11 (undekad). Dedicated software for this prediction has been developed, sich as TWISTER^29^ and COILS^30^.

It is well known that also electrostatic interaction is a very important feature for protein structure and function. Aspartate, for example, is often involved in the catalytic triad of active centres in enzymes. Positively and negatively charged amino acids can form salt bridges and, for example, stabilize a-helices when located at a distance of four positions in the chain.

Taylor^5^ proposed adjectives like ‘small’ and ‘hydrophobic’ to describe the features used. Each amino acid is then considered to be either small or not small, hydrophobic or non-hydrophobic, etc. Here, we suggest using the following adjectives. For volume and hydrophobicity, again, the terms ‘small’ and ‘hydrophobic’ are the obvious choice. Further, we propose ‘electrostatically interactive’ for amino acids with an Electron–Ion-Interaction-Potential higher than 0.044, ‘α-helix abundant’ when the α-helix propensity is higher than 1.01, ‘π-Helix abundant’ when the π-helix propensity is higher than 0.95.

It can be seen immediately that most of those features are very dissimilar to each other. There is no obvious connection between the volume of a substance and its hydrophobicity. Further, the propensities should be influenced by many indices, both physicochemical and biological and are therefore not expected to be strongly connected to just one of the other mentioned indices. The lack of correlation between α-helix propensity and π-helix-propensity is not as obvious, however.

For the vertical structure of the tree graph (Fig. 2) corresponding to a given set of five features, there are 5! = 120 arrangements (permutations of five features). Figure 2 shows only one out of these permutations. However, for a representation in terms of sets, i.e. the Euler diagram, these permutations do not matter.

While the two hydrophobic amino acids leucine and isoleucine are only separated in the fourth step (isoleucine has the lower α-helix propensity as it is branched closer towards the beginning of the side chain and therefore leads to detrimental sterical interactions in the α-helix), it might be surprising that the very similar amino acids glutamine and asparagine are separated in the first step already, and that arginine and tyrosine stay together up to the last step. The main result of the classification is the separation as represented by the Euler diagram rather than the tree graph. Nevertheless, one may try to find an optimal arrangement of the tree graph that fits better to the traditional classification, in which, for example, glutamine and asparagine or leucine and isoleucine stay together “as long as possible”. We leave this question to further studies.

In respect of our goal to develop suggestions which non-canonical amino acids to incorporate in experimental expansions of the genetic code within synthetic biology it is detrimental that neither α-helix propensity nor π-helix-propensity can be directly determined for non-canonical amino acids. On the other hand, for classification, it is sufficient to know whether the value is above or below the median. For some non-canonical amino acids such as butyrine, norvaline, and norleucine it is clear that they have a high α-helix propensity because they have unbranched side chains^31^. The method becomes more and more applicable in this direction as the knowledge about secondary structures of synthetic proteins increases. Moreover, this issue does not reduce the value of our method in comparison to Taylor’s method because that was not originally devised for non-canonical amino acids.

Moreover, we investigated if any of the other features showing a high degree of correlation with the two propensities allow an identical separation of the proteinogenic amino acids (Tables S3–S7). A list of highly correlated features can be directly retrieved from each feature entry at AAindex. Our hope was to identify features which might replace the propensities and can be easily determined for non-canonical amino acids. Unfortunately, for the π-helix-propensity there is only one highly correlated (|Correlation coefficient|> 0.8) feature (TANS770104—Normalized frequency of chain reversal R) which neither shows the same numerical ordering of the amino acids nor identical separation. There are 21 features that are mathematically highly correlated with α-helix propensity. However, most of them are just slight variations of the α-helix propensity and the only one of those features which exhibits identical separation (LEVM780101—Normalized frequency of alpha-helix, with weights) is completely identical to the α-helix propensity of our result.

We next searched the literature for experimental methods which are able to approximate the helix propensities based on protein evolution, for example by thermodynamic destabilization of short α-helical peptides upon incorporation of the respective amino acid. Such methods in principle also work for non-canonical amino acids. They are available for α-helix propensity^32^ but to our knowledge not for π-helix-propensity. Even if such methods are not perfectly accurate in predicting the propensities, as they only have to assign the non-canonical amino acid to one of the sides left or right of the median in our method for binary vector assignment, they should be sufficient in most cases.

Finally, we checked if upon replacement of the interpretability criterion with the criterion that the feature is predictable for non-canonical amino acids we are able to identify a solution with the minimum number of features. We retained 199 features of the database for search and retrieved 26,547 solutions. How to prioritize those solutions we leave for further research.

### Identification of more combinations by non-median based separation

Allowing for separations not necessarily based on the median allows for identification of new combinations with interesting properties, e.g. more meaningful separation points or features with higher immediate biological significance. One way of identifying more solutions is to increase the number of features used for classification. For example, one could allow for six features to increase the amount of solutions tremendously. Doing so, one may pick solutions containing only features which are relevant for the biological context at hand. However, as we want to use the minimum number of features for optimal classification, we aim for binary separation not only at the median but at any point between the 20 amino acids except for those resulting in an unresolvable group with more than 16 amino acids. Because allowing for multiple separation points for each feature might very quickly lead to a steeply increasing computational effort, one needs criteria for what particular separations to include in the search. Those criteria can be varied depending on the context of study at hand. This adds to the flexibility of our approach. For demonstration purposes we stick to the simple rule of including only the 3 separations of each feature which separate at the largest gaps after excluding separations which can not be part of a solution composed of the minimum amount of five features. Additionally, if two or more separations classify the amino acids identically, we only keep the separation with the highest score. The score of a particular separation is equal to the number of other separations for the same feature which separate between amino acids with a lower difference in the numerical value for the feature at hand. Therefore, the highest score a separation can obtain is 18, namely if it is the separation separating between adjacent amino acids farthest apart from each other.

**Figure 5:**
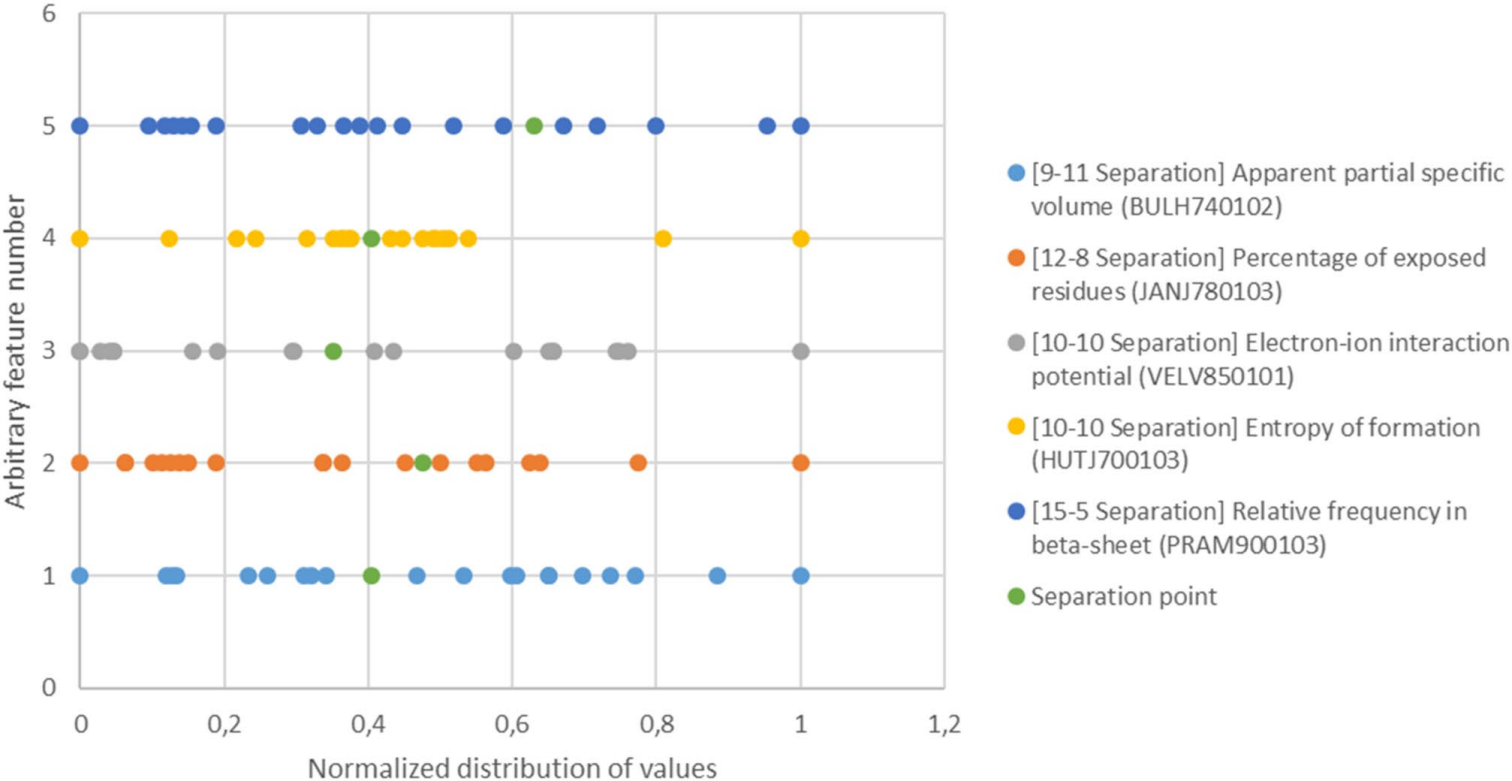
Highest scoring solution identified with the emphasis on how natural the separations of the composing features are. The x–y Separation in brackets means, that the particular feature separates into x amino acids in the group with small numerical values and y amino acids in the group with large numerical values for the particular feature. The numerical values have been normalized to depict all separations on a single diagram.

Figure 5 shows the highest scoring solution we identified. The cumulative score equals 76, which is quite close to the maximum score of 5 * 18 = 90. Interestingly, of the features included into the solution, there is again one volume-related (apparent partial specific volume,), one hydrophobicity-related (percentage of exposed residues,), one concerning electrostatic interactions (the same as in our previous solution, the electron–ion interaction potential.), and another one about abundance in secondary structures (relative frequency in beta-sheets.). But instead of another secondary structure related feature, this time the last feature is based on a concept from thermodynamics, namely the entropy of formation.

### Application and validation

Advancements in genetics have made it possible to introduce new pairs of tRNA/aminoacyl-tRNA synthetase into organisms to expand the genetic code with non-canonical amino acids, e.g. by changing the meaning of stop codons^33^. This opened up the question of what non-canonical amino acids to choose for genetic code expansion. In some contexts, this question is narrowed down by the purpose of the experiment itself. For example, if one wants to incorporate an amino acid for bio-orthogonal labeling, one has to choose from a narrow pool of amino acids containing alkyne or analogous moieties as a bio-orthogonal handle^34,35^. There exist other contexts, however, where incorporating and therefore identifying beforehand an amino acid that is functionally and structurally as different as possible from the canonical set of amino acids is the main challenge, all the more as the specificity of aminoacyl tRNA synthetases needs to be changed. This diversity can be assessed by counting how often the non-canonical amino acid occupies empty vectors in our solutions.

**Figure 6:**
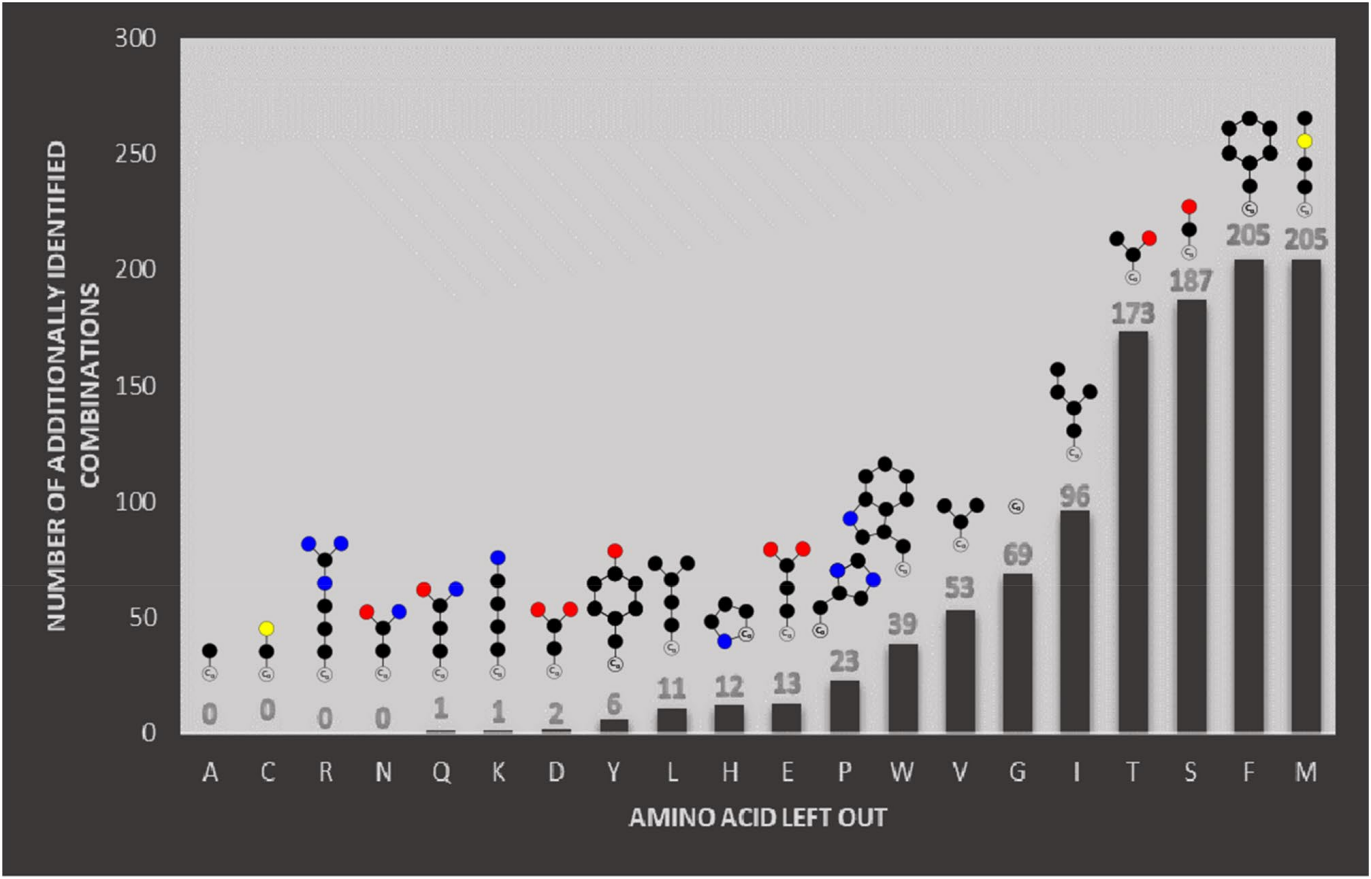
Diagram showing the contribution of each amino acid to redundancy, assessed by identifying how many optimal classifications can be achieved additionally if one amino acid is left out during the search.

Another interesting question is how much the number of solutions for median-based classification increases if we consider only a set of 19 out of the 20 proteinogenic amino acids. The amino acid which leads to the largest increase in identified combinations should be the most redundant one as it most often occupies a common vector with another amino acid whereas all the other vectors are occupied by one single amino acid at most. The results of the corresponding calculations are shown in Fig. 6. One can immediately see, that there are huge differences between the amino acid redundancy scores. It appears that amino acids with a lower number of functional groups in general score higher, which makes sense as they lack the means to deviate in their characteristics from other hydrophobic amino acids and are therefore more redundant. Also, serine and threonine rank relatively high, probably due to their structural similarity. Though this reasoning seems not to apply to the very similar amino acids, glutamine, asparagine, glutamate, and aspartate. This could be due to the coherence of the remaining three amino acids which prevent the identification of new combinations as far as only one of them is not considered during the search.

## Conclusion

We proposed a novel method for selecting groups of five features that enable an optimal classification of the 20 proteinogenic amino acids based on binary separation. To discover meaningful combinations of features, we manually selected 83 features out of the 566 features occurring in the AAindex database^18–20^. Solely using median-based separation, we were able to discover 18 combinations, although three features were present in every single combination. By plausible additional criteria, we came up with the following five properties: small, hydrophobic, electrostatically interactive, α-Helix abundant, π-Helix abundant. Thus, an easily understandable and interpretable classification scheme with a minimum number of features has been found. For example, it can help biochemistry students to become familiar with the properties of amino acids.

We additionally discovered a high-ranking combination by allowing for separation other than by the median and selecting only those separations with natural points of separation. Our method can predict how distinct a non-canonical amino acid is compared to the set of 20 proteinogenic amino acids by counting how often that amino acid occupies empty vectors in the identified combinations and therefore assumes a niche unoccupied by any of the proteinogenic amino acids. The prerequisite for this procedure is that the values of the non-canonical amino acid are known for the features that the solution consists of. It is worth noting that using five features, we can classify 32 amino acids, at least 12 of which then could be non-canonical. By using six features, we could even classify 64 amino acids.

Choosing which features to be included from the AAindex database can adjust the method to specific contexts. As genetic methods enable the introduction of non-canonical amino acids into the genetic code, this method may assist in deciding which of these amino acids to choose if the task is to improve overall diversity. Each amino acid fulfills one or several biological functions and can often (but not under all circumstances) be replaced by amino acids with similar properties^36^. Our classification system could prove helpful in predicting such replacements.

A drawback of our suggested classification is that some of the adjectives we assign to the amino acids in specific groups are not as intuitive as those used by Taylor^5^. While in Taylor’s classification, adjectives like “small”, “charged” are easy to grasp, there are some adjectives in our classification like α-Helix abundant which need further clarification to be understood. However, it is also an interesting insight, that despite of volume-, hydrophobicity-, and electrostatics-related features the solution contains also more complex features related to secondary structure abundance or thermodynamics. Especially as the same general composition of features can also be seen in our solution focused on the natural group separation by scoring non-median based separations. The amount of solutions found improves tremendously upon including non-median based separations. This allows for the flexibility to develop own criteria to identify solutions ultimately suited for the description of amino acids in the particular context of research.

## Supplementary information

Supplementary Information.
