# Supplementary material for: A novel method for achieving an optimal classification of the proteinogenic amino acids
Source: Sci Rep. 2020 Sep 18;10:15321. doi: 10.1038/s41598-020-72174-5 (PMC7501307; doi:10.1038/s41598-020-72174-5)
Supplement: Supplementary file 1 — Supplementary Information. [file 41598_2020_72174_MOESM1_ESM.pdf]

A novel method for achieving an optimal classification of the proteinogenic amino acids

supplementary information

Andre Then[1,a], Karel Mácha[1,2,a], Bashar Ibrahim[1,*], Stefan Schuster[1,*]

[1] Chair of Bioinformatics, Matthias Schleiden Institute, University of Jena, Ernst-Abbe-Platz 2, Jena 07743, Germany. andre.then@uni-jena.de; bashar.ibrahim@uni-jena.de; stefan.schu@uni-jena.de.

[2] Present address: Westernacher Solutions, Columbiadamm 37, Berlin 10965, Germany. karel.macha@karlitos.de.

[a] These authors contributed equally to this work

* Corresponding authors

S1

ID	Description
ARGP820101	Hydrophobicity index (Argos et al., 1982)
BIGC670101	Residue volume (Bigelow, 1967)
BULH740102	Apparent partial specific volume (Bull-Breese, 1974)
BURA740101	Normalized frequency of alpha-helix (Burgess et al., 1974)
CHAM820102	Free energy of solution in water, kcal/mole (Charton-Charton, 1982)
CHAM830103	The number of atoms in the side chain labelled 1+1 (Charton-Charton, 1983)
CHAM830104	The number of atoms in the side chain labelled 2+1 (Charton-Charton, 1983)
CHAM830105	The number of atoms in the side chain labelled 3+1 (Charton-Charton, 1983)
CHAM830106	The number of bonds in the longest chain (Charton-Charton, 1983)
CHOC750101	Average volume of buried residue (Chothia, 1975)
CHOC760101	Residue accessible surface area in tripeptide (Chothia, 1976)
CHOC760102	Residue accessible surface area in folded protein (Chothia, 1976)
CHOC760104	Proportion of residues 100% buried (Chothia, 1976)
CHOP780201	Normalized frequency of alpha-helix (Chou-Fasman, 1978b)
CHOP780202	Normalized frequency of beta-sheet (Chou-Fasman, 1978b)
CIDH920105	Normalized average hydrophobicity scales (Cid et al., 1992)
COHE430101	Partial specific volume (Cohn-Edsall, 1943)
DAWD720101	Size (Dawson, 1972)
EISD840101	Consensus normalized hydrophobicity scale (Eisenberg, 1984)
EISD860101	Solvation free energy (Eisenberg-McLachlan, 1986)
FASG760101	Molecular weight (Fasman, 1976)
FASG760102	Melting point (Fasman, 1976)
FASG760103	Optical rotation (Fasman, 1976)
FAUJ880104	STERIMOL length of the side chain (Fauchere et al., 1988)
FAUJ880105	STERIMOL minimum width of the side chain (Fauchere et al., 1988)
FAUJ880106	STERIMOL maximum width of the side chain (Fauchere et al., 1988)
FAUJ880109	Number of hydrogen bond donors (Fauchere et al., 1988)
FAUJ880110	Number of full nonbonding orbitals (Fauchere et al., 1988)
FAUJ880111	Positive charge (Fauchere et al., 1988)
FAUJ880112	Negative charge (Fauchere et al., 1988)
GOLD730101	Hydrophobicity factor (Goldsack-Chalifoux, 1973)
GOLD730102	Residue volume (Goldsack-Chalifoux, 1973)
GRAR740102	Polarity (Grantham, 1974)
GRAR740103	Volume (Grantham, 1974)
HOPT810101	Hydrophilicity value (Hopp-Woods, 1981)
HUTJ700101	Heat capacity (Hutchens, 1970)
HUTJ700102	Absolute entropy (Hutchens, 1970)
HUTJ700103	Entropy of formation (Hutchens, 1970)
JANJ780101	Average accessible surface area (Janin et al., 1978)
JANJ780102	Percentage of buried residues (Janin et al., 1978)
JANJ780103	Percentage of exposed residues (Janin et al., 1978)
JANJ790101	Ratio of buried and accessible molar fractions (Janin, 1979)
JOND750101	Hydrophobicity (Jones, 1975)
JOND920101	Relative frequency of occurrence (Jones et al., 1992)
KLEP840101	Net charge (Klein et al., 1984)
KRIW790102	Fraction of site occupied by water (Krigbaum-Komoriya, 1979)
KRIW790103	Side chain volume (Krigbaum-Komoriya, 1979)
LEVM760101	Hydrophobic parameter (Levitt, 1976)
LEVM760102	Distance between C-alpha and centroid of side chain (Levitt, 1976)

LEVM760105	Radius of gyration of side chain (Levitt, 1976)
MCMT640101	Refractivity (McMeekin et al., 1964), Cited by Jones (1975)
PRAM900101	Hydrophobicity (Prabhakaran, 1990)
PRAM900102	Relative frequency in alpha-helix (Prabhakaran, 1990)
PRAM900103	Relative frequency in beta-sheet (Prabhakaran, 1990)
RACS770103	Side chain orientational preference (Rackovsky-Scheraga, 1977)
RADA880106	Accessible surface area (Radzicka-Wolfenden, 1988)
RADA880107	Energy transfer from out to in(95%buried) (Radzicka-Wolfenden, 1988)
RADA880108	Mean polarity (Radzicka-Wolfenden, 1988)
ROBB790101	Hydration free energy (Robson-Osguthorpe, 1979)
ROSM880101	Side chain hydropathy, uncorrected for solvation (Roseman, 1988)
VELV850101	Electron-ion interaction potential (Veljkovic et al., 1985)
VENT840101	Bitterness (Venanzi, 1984)
VHEG790101	Transfer free energy to lipophilic phase (von Heijne-Blomberg, 1979)
WARP780101	Average interactions per side chain atom (Warme-Morgan, 1978)
WERD780101	Propensity to be buried inside (Wertz-Scheraga, 1978)
WOLR810101	Hydration potential (Wolfenden et al., 1981)
ZIMJ680101	Hydrophobicity (Zimmerman et al., 1968)
ZIMJ680102	Bulkiness (Zimmerman et al., 1968)
ZIMJ680103	Polarity (Zimmerman et al., 1968)
ZIMJ680104	Isoelectric point (Zimmerman et al., 1968)
FODM020101	Propensity of amino acids within pi-helices (Fodje-Al-Karadaghi, 2002)
MITS020101	Amphiphilicity index (Mitaku et al., 2002)
PONP930101	Hydrophobicity scales (Ponnuswamy, 1993)
KUHL950101	Hydrophilicity scale (Kuhn et al., 1995)
ZHOH040103	Buriability (Zhou-Zhou, 2004)
PONJ960101	Average volumes of residues (Pontius et al., 1996)
WOLR790101	Hydrophobicity index (Wolfenden et al., 1979)
OLSK800101	Average internal preferences (Olsen, 1980)
BLAS910101	Scaled side chain hydrophobicity values (Black-Mould, 1991)
CASG920101	Hydrophobicity scale from native protein structures (Casari-Sippl, 1992)
FASG890101	Hydrophobicity index (Fasman, 1989)
KARS160101	Number of vertices (order of the graph) (Karkbara-Knisley, 2016)
KARS160102	Number of edges (size of the graph) (Karkbara-Knisley, 2016)

S2

ID	Description	Nr. missing values	AA-Code for amino acids with missing values
CHAM820102	Free energy of solution in water, kcal/mole (Charton-Charton, 1982)	4	N H K T
GOLD730101	Hydrophobicity factor (Goldsack-Chalifoux, 1973)	2	N Q
RADA880107	Energy transfer from out to in(95%buried) (Radzicka-Wolfenden, 1988	1	P
RADA880108	Mean polarity (Radzicka-Wolfenden, 1988)	1	P

S3

Result_feature	Correlated_feature	CC	Description	identical separation?	identical order?
GRAR740103	BIGC670101	0.984	Residue volume (Bigelow, 1967)	TRUE	FALSE
GRAR740103	GOLD730102	0.984	Residue volume (Goldsack-Chalifoux, 1973)	TRUE	FALSE
GRAR740103	TSAJ990101	0.979	Volumes including the crystallographic waters using the ProtOr (Tsai et al., 1999)	TRUE	FALSE
GRAR740103	TSAJ990102	0.978	Volumes not including the crystallographic waters using the ProtOr (Tsai et al., 1999)	TRUE	FALSE
GRAR740103	CHOC750101	0.973	Average volume of buried residue (Chothia, 1975)	TRUE	FALSE
GRAR740103	FAUJ880103	0.959	Normalized van der Waals volume (Fauchere et al., 1988)	TRUE	FALSE
GRAR740103	CHAM820101	0.951	Polarizability parameter (Charton-Charton, 1982)	TRUE	FALSE
GRAR740103	HARY940101	0.946	Mean volumes of residues buried in protein interiors (Harpaz et al., 1994)	TRUE	FALSE
GRAR740103	CHOC760101	0.945	Residue accessible surface area in tripeptide (Chothia, 1976)	FALSE	FALSE
GRAR740103	PONJ960101	0.937	Average volumes of residues (Pontius et al., 1996)	TRUE	FALSE
GRAR740103	ROSG850101	0.922	Mean area buried on transfer (Rose et al., 1985)	FALSE	FALSE
GRAR740103	RADA880106	0.92	Accessible surface area (Radzicka-Wolfenden, 1988)	FALSE	FALSE
GRAR740103	RADA880106	0.92	Accessible surface area (Radzicka-Wolfenden, 1988)	FALSE	FALSE
GRAR740103	FASG760101	0.908	Molecular weight (Fasman, 1976)	FALSE	FALSE
GRAR740103	LEVM760105	0.9	Radius of gyration of side chain (Levitt, 1976)	FALSE	FALSE
GRAR740103	CHAM830106	0.89	The number of bonds in the longest chain (Charton-Charton, 1983)	FALSE	FALSE
GRAR740103	LEVM760102	0.885	Distance between C-alpha and centroid of side chain (Levitt, 1976)	FALSE	FALSE
GRAR740103	ZHOH040102	0.872	The relative stability scale extracted from mutation experiments (Zhou-Zhou, 2004)	FALSE	FALSE
GRAR740103	DAWD720101	0.853	Size (Dawson, 1972)	FALSE	FALSE
GRAR740103	LEVM760106	0.846	van der Waals parameter R0 (Levitt, 1976)	FALSE	FALSE
GRAR740103	LEVM760107	0.841	van der Waals parameter epsilon (Levitt, 1976)	FALSE	FALSE
GRAR740103	FAUJ880106	0.819	STERIMOL maximum width of the side chain (Fauchere et al., 1988)	FALSE	FALSE
GRAR740103	MCMT640101	0.817	Refractivity (McMeekin et al., 1964), Cited by Jones (1975)	FALSE	FALSE
GRAR740103	RADA880103	-0.881	Transfer free energy from vap to chx (Radzicka-Wolfenden, 1988)	FALSE	FALSE

S4

Result_feature	Correlated_feature	CC	Description	identical separation?	identical order?
VELV850101	COSI940101	1.000	Electron-ion interaction potential values (Cosic, 1994)	TRUE	TRUE

S5

Result_feature	Correlated_feature	CC	Description	identical separation?	identical order?
PRAM900101	ENGD860101	1.000	Hydrophobicity index (Engelman et al., 1986)	TRUE	TRUE
PRAM900101	ROSM880101	0.917	Side chain hydropathy, uncorrected for solvation (Roseman, 1988)	FALSE	FALSE
PRAM900101	VHEG790101	0.909	Transfer free energy to lipophilic phase (von Heijne-Blomberg, 1979)	FALSE	FALSE
PRAM900101	KUHL950101	0.908	Hydrophilicity scale (Kuhn et al., 1995)	FALSE	FALSE
PRAM900101	OOBM770101	0.907	Average non-bonded energy per atom (Oobatake-Ooi, 1977)	TRUE	FALSE
PRAM900101	JANJ780101	0.901	Average accessible surface area (Janin et al., 1978)	FALSE	FALSE
PRAM900101	ROSM880102	0.892	Side chain hydropathy, corrected for solvation (Roseman, 1988)	FALSE	FALSE
PRAM900101	PUNT030101	0.889	dge-based membrane-propensity scale from 1D_Helix in MPtopo databases (Punta-Marita	FALSE	FALSE
PRAM900101	JANJ780103	0.884	Percentage of exposed residues (Janin et al., 1978)	FALSE	FALSE
PRAM900101	HOPT810101	0.881	Hydrophilicity value (Hopp-Woods, 1981)	FALSE	FALSE
PRAM900101	GUYH850104	0.881	Apparent partition energies calculated from Janin index (Guy, 1985)	FALSE	FALSE
PRAM900101	LEVM760101	0.881	Hydrophobic parameter (Levitt, 1976)	FALSE	FALSE
PRAM900101	WOEC730101	0.871	Polar requirement (Woese, 1973)	FALSE	FALSE
PRAM900101	PUNT030102	0.869	dge-based membrane-propensity scale from 3D_Helix in MPtopo databases (Punta-Marita	FALSE	FALSE
PRAM900101	GUYH850105	0.867	Apparent partition energies calculated from Chothia index (Guy, 1985)	FALSE	FALSE
PRAM900101	KIDA850101	0.866	Hydrophobicity-related index (Kidera et al., 1985)	FALSE	FALSE
PRAM900101	GRAR740102	0.855	Polarity (Grantham, 1974)	FALSE	FALSE
PRAM900101	ZIMJ680103	0.854	Polarity (Zimmerman et al., 1968)	FALSE	FALSE
PRAM900101	CHOC760102	0.826	Residue accessible surface area in folded protein (Chothia, 1976)	FALSE	FALSE
PRAM900101	MONM990101	0.82	Turn propensity scale for transmembrane helices (Monne et al., 1999)	FALSE	FALSE
PRAM900101	GUYH850101	0.82	Partition energy (Guy, 1985)	FALSE	FALSE
PRAM900101	FAUJ880109	0.815	Number of hydrogen bond donors (Fauchere et al., 1988)	FALSE	FALSE
PRAM900101	RADA880104	-0.803	Transfer free energy from chx to oct (Radzicka-Wolfenden, 1988)	FALSE	FALSE
PRAM900101	OLSK800101	-0.806	Average internal preferences (Olsen, 1980)	FALSE	FALSE
PRAM900101	CHOC760103	-0.814	Proportion of residues 95% buried (Chothia, 1976)	TRUE	FALSE
PRAM900101	NADH010103	-0.815	e based on self-information values in the two-state model (16% accessibility) (Naderi-Mar	FALSE	FALSE
PRAM900101	WARP780101	-0.827	Average interactions per side chain atom (Warme-Morgan, 1978)	FALSE	FALSE
PRAM900101	EISD860103	-0.831	Direction of hydrophobic moment (Eisenberg-McLachlan, 1986)	FALSE	FALSE
PRAM900101	NADH010101	-0.843	le based on self-information values in the two-state model (5% accessibility) (Naderi-Man	FALSE	FALSE
PRAM900101	KYTJ820101	-0.85	Hydropathy index (Kyte-Doolittle, 1982)	FALSE	FALSE
PRAM900101	FAUJ830101	-0.853	Hydrophobic parameter pi (Fauchere-Pliska, 1983)	FALSE	FALSE
PRAM900101	JANJ780102	-0.86	Percentage of buried residues (Janin et al., 1978)	FALSE	FALSE
PRAM900101	EISD860101	-0.862	Solvation free energy (Eisenberg-McLachlan, 1986)	FALSE	FALSE
PRAM900101	JURD980101	-0.862	Modified Kyte-Doolittle hydrophobicity scale (Juretic et al., 1998)	FALSE	FALSE
PRAM900101	BLAS910101	-0.864	Scaled side chain hydrophobicity values (Black-Mould, 1991)	FALSE	FALSE
PRAM900101	RADA880107	-0.865	Energy transfer from out to in(95%buried) (Radzicka-Wolfenden, 1988)	FALSE	FALSE
PRAM900101	NADH010102	-0.87	le based on self-information values in the two-state model (9% accessibility) (Naderi-Man	FALSE	FALSE
PRAM900101	WOLR790101	-0.877	Hydrophobicity index (Wolfenden et al., 1979)	FALSE	FALSE
PRAM900101	WOLR810101	-0.887	Hydration potential (Wolfenden et al., 1981)	FALSE	FALSE
PRAM900101	JANJ790102	-0.89	Transfer free energy (Janin, 1979)	FALSE	FALSE
PRAM900101	DESM900102	-0.89	Average membrane preference: AMP07 (Degli Esposti et al., 1990)	TRUE	FALSE
PRAM900101	ROSM880105	-0.912	Hydropathies of amino acid side chains, pi-values in pH 7.0 (Roseman, 1988)	FALSE	FALSE
PRAM900101	RADA880101	-0.932	Transfer free energy from chx to wat (Radzicka-Wolfenden, 1988)	FALSE	FALSE
PRAM900101	EISD840101	-0.936	Consensus normalized hydrophobicity scale (Eisenberg, 1984)	FALSE	FALSE
PRAM900101	JACR890101	-0.948	Weights from the IFH scale (Jacobs-White, 1989)	FALSE	FALSE

S6

Result_feature	Correlated_feature	CC	Description	identical separation?	identical order?
PRAM900102	LEVM780101	1.000	Normalized frequency of alpha-helix, with weights (Levitt, 1978)	TRUE	TRUE
PRAM900102	LEVM780104	0.964	Normalized frequency of alpha-helix, unweighted (Levitt, 1978)	FALSE	FALSE
PRAM900102	PALJ810101	0.943	Normalized frequency of alpha-helix from LG (Palau et al., 1981)	FALSE	FALSE
PRAM900102	KANM800101	0.942	Average relative probability of helix (Kanehisa-Tsong, 1980)	FALSE	FALSE
PRAM900102	ISOY800101	0.929	Normalized relative frequency of alpha-helix (Isogai et al., 1980)	FALSE	FALSE
PRAM900102	MAXF760101	0.924	Normalized frequency of alpha-helix (Maxfield-Scheraga, 1976)	FALSE	FALSE
PRAM900102	ROBB760101	0.916	Information measure for alpha-helix (Robson-Suzuki, 1976)	FALSE	FALSE
PRAM900102	GEIM800101	0.912	Alpha-helix indices (Geisow-Roberts, 1980)	FALSE	FALSE
PRAM900102	GEIM800104	0.907	Alpha-helix indices for alpha/beta-proteins (Geisow-Roberts, 1980)	FALSE	FALSE
PRAM900102	RACS820108	0.904	Average relative fractional occurrence in AR(i-1) (Rackovsky-Scheraga, 1982)	FALSE	FALSE
PRAM900102	PALJ810102	0.902	Normalized frequency of alpha-helix from CF (Palau et al., 1981)	FALSE	FALSE
PRAM900102	PALJ810109	0.898	Normalized frequency of alpha-helix in alpha/beta class (Palau et al., 1981)	FALSE	FALSE
PRAM900102	NAGK730101	0.894	Normalized frequency of alpha-helix (Nagano, 1973)	FALSE	FALSE
PRAM900102	CRAJ730101	0.887	Normalized frequency of middle helix (Crawford et al., 1973)	FALSE	FALSE
PRAM900102	CHOP780201	0.873	Normalized frequency of alpha-helix (Chou-Fasman, 1978b)	FALSE	FALSE
PRAM900102	TANS770101	0.854	Normalized frequency of alpha-helix (Tanaka-Scheraga, 1977)	FALSE	FALSE
PRAM900102	KANM800103	0.85	Average relative probability of inner helix (Kanehisa-Tsong, 1980)	FALSE	FALSE
PRAM900102	QIAN880107	0.829	Weights for alpha-helix at the window position of 0 (Qian-Sejnowski, 1988)	FALSE	FALSE
PRAM900102	QIAN880106	0.827	Weights for alpha-helix at the window position of -1 (Qian-Sejnowski, 1988)	FALSE	FALSE
PRAM900102	BURA740101	0.805	Normalized frequency of alpha-helix (Burgess et al., 1974)	FALSE	FALSE
PRAM900102	NAGK730103	-0.809	Normalized frequency of coil (Nagano, 1973)	FALSE	FALSE

S7

Result_feature	Correlated_feature	CC	Description	identical separation?	identical order?
FODM020101	TANS770104	-0.802	Normalized frequency of chain reversal R (Tanaka-Scheraga, 1977)	FALSE	FALSE
